# “It must start with me, so it started with me”: A qualitative study of Project YES! youth peer mentor implementing experiences supporting adolescents and young adults living with HIV in Ndola, Zambia

**DOI:** 10.1371/journal.pone.0261948

**Published:** 2022-02-03

**Authors:** Virginia M. Burke, Christiana Frimpong, Sam Miti, Jonathan K. Mwansa, Elizabeth A. Abrams, Katherine G. Merrill, Julie A. Denison

**Affiliations:** 1 Department of International Health, Johns Hopkins Bloomberg School of Public Health, Baltimore, Maryland, United States of America; 2 Arthur Davison Children’s Hospital, Ndola, Zambia; Emory University School of Medicine, UNITED STATES

## Abstract

**Background:**

Little is known about youth-led approaches to addressing HIV-related outcomes among adolescents and young adults (AYA) living with HIV. In response, Project YES! hired and trained youth living with HIV as peer mentors (YPMs) in four HIV clinics in Ndola, Zambia to hold meetings with 276 15-24-year-olds living with HIV. Within this randomized controlled trial, a qualitative sub-study was conducted to explore YPMs’ implementing experiences.

**Methods:**

In-depth interviews were conducted with the eight YPMs (50% female) ages 21–26 years. YPMs were asked about their experiences working with clients, their feedback on program components, and what the experience meant to them personally and professionally. Interviews were audio-recorded, transcribed verbatim, and thematic analysis was performed.

**Results:**

YPMs connected with AYA clients by discussing shared struggles, modeling positive health behaviors, and establishing judgement-free environments. YPMs experienced powerful personal transformations in HIV-related health behaviors, conceptions of self, and plans for the future. Many expressed now seeing themselves as community leaders–“ambassadors”, “game changers”–and “not just alone in this world.” They described newfound commitments to reaching personal and professional goals. YPMs were adamant that Project YES! should expand so other HIV-positive AYA might benefit.

**Conclusion:**

Well-trained and compensated YPMs who are integrated into HIV clinics can support AYA in unique and important ways due to their shared experiences. The transformational experience of becoming YPMs empowers youth to see themselves as role models and leaders. Future programs should engage youth living with HIV as partners in efforts to end the HIV epidemic.

## Introduction

An estimated 1.6 million adolescents, between the ages of 10 to 19 years, are living with HIV globally [1]. The severity of HIV infection is often measured by viral load, or the number of RNA copies in one milliliter of blood [2]. Lower viral load is associated with decreased disease progression and risk of transmission [3,4]. In Zambia, a country with one of the world’s highest HIV prevalence rates among the general population, only a third of adolescents living with HIV are estimated to virally suppressed (defined as ≤1000 copies/mL) compared to three-quarters of adults [5].

Several factors increase adolescent and young adult (AYA) vulnerability. Past studies have shown that AYA living with HIV struggle with clinic attendance, adherence to antiretroviral therapy (ART), and viral suppression (VS) compared to adults [6–12]. AYA report significant experiences of stigma and discrimination based on their HIV status, contributing to these poor outcomes [13–15]. These challenges are further complicated by the fact that AYA are in a unique life stage of rapid physical and psychological development [16,17]. The resulting disparities in HIV-related outcomes reflect a well-established need for accessible, AYA-specific HIV programming that addresses the needs of young people [7,9,18–21] and involves AYA in the design and implementation of interventions [19,21–23].

In response, we designed and implemented Project YES! Youth Engaging for Success, a randomized controlled trial testing the impact of a peer mentoring intervention among 15-24-year-olds living with HIV in Ndola, Zambia on VS. Within this trial, a qualitative in-depth sub-study informed by an implementation science approach was conducted to explore Youth Peer Mentor (YPM) experiences–challenges and successes–implementing Project YES!. Interviews aimed to assess perceived acceptability and feasibility of the intervention from the YPM perspective. These data are critical to understanding how a YPM program works and to inform expansion and scale-up of a youth-led, adult-supported, effective intervention.

### Project YES! overview

Project YES! participants (n = 276), ages 15–24 years, were consecutively recruited from four peri-urban study sites in Ndola, Zambia, including a children’s hospital (pediatric setting), an adult hospital (adult setting), and two primary care facilities (adult settings). Youth were recruited at both pediatric and adult clinics because at the time of the study there were only two pediatric hospitals in Zambia and the majority of youth living with HIV received services at clinics that were not developed nor tailored to serve young people. As described in the primary paper, the two hospitals had HIV clinics with adolescent-focused days and hours, while the two primary care clinics offered HIV services on specific days [24]. Enrolled participants were randomized to either an intervention arm or comparison arm (ClinicalTrials.gov NCT04115813) [24]. The intervention consisted of one orientation meeting, six monthly one-on-one meetings with an assigned YPM, and six monthly youth group meetings facilitated by YPMs. Youth participants were able to invite a caregiver to attend their orientation meeting and three optional caregiver group meetings over the six-month intervention. YPMs and clients communicated in Bemba and English, depending on client preference. Results from the primary analysis of this trial have been detailed elsewhere. In brief, Project YES! participants experienced a reduction in internalized stigma by a factor of 0.39 [interaction term odds ratio (OR):0.39, 95% confidence interval (CI):0.21,0.73] in the intervention arm relative to the reduction in the comparison arm. In a sub-analysis among participants in the pediatric setting, Project YES! also found a relative increase in the odds of VS by a factor of 4.7 [interaction term OR: 4.66, 95% CI: 1.84–11.78] among intervention compared to comparison group participants [24]. Based on these data, the CDC included Project YES! in the Compendium of Evidence-Based Interventions (EBI) and Best Practices for HIV Prevention [25].

YPMs were selected by Health Care Providers (HCPs) at each study clinic and were slightly older (ages 21–26 years old) than their AYA clients, living with HIV, and perceived by the HCP to have successfully transitioned to HIV self-management. Project YES! YPMs received payment commensurate with that of other lay health workers at the clinics. For most YPMs, Project YES! was their first paid professional position. Before the start of the intervention, YPMs underwent a capacity-building process, starting with an intensive two-week pre-service training led by a Training and Capacity Building Specialist (TCBS). This pre-service training included opportunities for YPMs to reflect on their own health behaviors and experiences living with HIV and share these experiences in group discussions facilitated by the TCBS. Second, YPMs had one month of practice meetings with non-study youth living with HIV (18 years and older) prior to the start of the intervention. Third, midway through Project YES! implementation, YPMs underwent an in-service training with the same TCBS to review experiences and reinforce mentoring skills. Fourth, YPMs met weekly as a group to discuss challenges, approaches, and ideas with study leads. Lastly, study team members provided ongoing supportive supervision throughout study implementation to respond to YPM needs in real time. With the exception of one who moved out of town, all hired YPMs remained throughout the intervention.

## Methods

### Ethics approval

This study was reviewed and approved by the ERES Converge Institutional Review Board in Zambia, the Zambia Ministry of Health through the National Health Research Authority, and the Johns Hopkins Bloomberg School of Public Health Institutional Review Board in the United States. YPMs provided written informed consent for participation prior to the start of the in-depth interviews.

### Qualitative data collection & analysis

This qualitative inquiry was informed by the CFIR implementation science approach [26] to explore YPMs’ experiences as facilitators of Project YES!. Much of the inquiry focused on the “dynamic interplay” described within the CFIR framework that characterizes the interdependent relationship between individual YPM implementers and the Project YES! program itself. Interview guides were used to focus discussions on YPM experiences working with clients, their feedback on program components, and what the experience meant to them personally and professionally. Probes encouraged participants to share both positive and negative experiences, how it felt to discuss sensitive issues such as sex and alcohol use with clients, and recommendations for future programs, among others. A trained interviewer (VMB) conducted semi-structured, in-depth interviews with all eight Project YES! YPMs November 4–17, 2018. All Project YES! YPMs were invited for an interview and all accepted. Participants were 50% female, ages 21–26 years old, and living with HIV. Each YPM participated in a single interview that lasted 1–2 hours (average 1hr 40 min). The interviewer (VMB) had extensive qualitative research training and had been working with this group of YPMs since the start of Project YES! as a Research Associate. In her position, she developed a trusting relationship with the YPMs which fostered open communication during interviews. The author encouraged participants to share both positive and negative feedback about their experiences so that it could be improved for the future. Interviews were conducted in a private space at either the participant’s workplace or home, depending on preference and availability, in English, audio-recorded, and transcribed verbatim.

Audio files were transcribed by a company in Lusaka, edited for accuracy by the interviewer (VMB), and imported into NVivo 12 for analysis. Transcripts were de-identified and numbered. Data were analyzed iteratively through deductive and inductive coding. The primary author (VMB) developed an initial codebook based on original research questions and revised codes as needed with co-authors (KGM, EA, JAD). Deductive coding focused on summarizing feedback on program elements; inductive coding identified themes that emerged within YPM responses. Summarized results were shared with participants during dissemination activities to confirm accuracy of interpretation.

## Results

Two main themes emerged from across the in-depth interviews with the eight YPMs, illustrating the interconnectedness of individual behavior change and organizational impact. First, all YPMs described personal transformations over the course of the project in their own HIV-related health behaviors, conceptions of self, and plans for the future. These changes were closely tied to a shared sense of responsibility to model positive behaviors for their youth clients. Secondly, YPMs emphasized the importance of using their shared experiences as AYA living with HIV, combined with their new mentoring skills, to connect, build trust, and link clients to needed care. Each of these themes is detailed below:

### “It’s my life”: YPM personal transformations in their own HIV-related health behaviors, conceptions of self, and plans for the future

YPMs characterized their introduction to Project YES!–the pre-service training (PST) and practice sessions–as the start of a period of heightened learning, personal reflection, and group bonding. Many YPMs appreciated understanding how ART works in the body at a more detailed level and *why* it is important to take the drugs at the same time every day–“it made me change in a certain way that if I don’t take my medication… I will face the consequences.”

The pre-service training was also a time for the YPMs to reflect within a group setting on the challenges they have experienced in their own lives. This was the first time many of these youth had shared their experiences with a group, providing an opportunity to work through difficult issues with their peers and the TCBS, which they reported enabled them to better support their future clients. One YPM recalled a particularly emotional training session about loss and grief:

*As peer mentors, we looked at healing for loss and grief and we cried. [The TCBS] talked about it. …We even had a chance to share even to our fellow YPMs… so that in case you meet that [issue] in the future …you meet it, you will know how to come about it or how to handle that situation… It really helped. It really prepared us and quickly. (Male YPM)*

This period of growth and self-reflection only intensified with regular meetings with their AYA clients. Many YPMs discussed a shift in their views of the world and of themselves from “negative” to “positive thinking,” reframing the narrative of their lives and their futures from one of victimization to one of strength and the ability to help others. Two YPMs referred to themselves as “game changers” with the potential to have positive impacts on the world around them:

*Sometimes I used to pity myself, self-pity. But now, I’m a positive thinker and I’m a game changer. I can change someone’s game! <laughs> … like if someone thinks that they cannot go high, I can change their thinking that, my dear, you can go high, if only you can have determination. (Female YPM)*

All YPMs expressed a sense of duty to be positive role models for their clients. Some YPMs explicitly mentioned the importance of appearing well-kept and healthy to show clients what is possible if they maintain good adherence. They also emphasized modeling positive behaviors in the community as well as the health facilities so as not to undermine the messages they were trying to promote. This mentality encouraged YPMs to consistently practice positive health behaviors in their own lives, particularly behaviors related to addressing adherence and stigma. As one YPM shared:

*If I am dealing with stigma in a negative way, what am I going to tell to my clients? How am I going to mentor about stigma to my clients? So firstly when I started Project YES I had to take it personally. Like, if I want to be a mentor to that client helping that client suppress his or her viral load, it must start with me. I must follow the topics that we have, and I must put it to practice. If I want that person to have a non-detectable viral load, it must start with me, so it started with me. (Female YPM)*

Throughout YPM interviews, informants expressed how they had changed since becoming peer mentors with words like “courage” and “strength”. YPMs frequently referenced learning from their clients, even as they were teaching them. Many described a new sense of ownership over their own HIV management and lives–“it’s my life”–after working with Project YES!. One YPM proudly shared that he is now the one who reminds his own mother to take her medications, rather than the other way around. Several shared that they felt less vulnerable to stigma. One YPM triumphantly described how she is no longer afraid of people knowing her status:

*At first I never used to like it when someone even talks about my status. I used to feel like beating that person, but right now I don’t care. It’s like I have learned to understand more about disclosure and about how I can deal with stigma, yes, all those areas. At first if someone discriminates me I used to feel bad, but I know how to handle it in a positive way … and I can even stand in front of the whole world and say "This is who I am. I am not afraid, and I’m not ashamed. This is who I am.” (Female YPM)*

Becoming YPMs also shifted informants’ perspectives on the future. Four distinct sub-themes emerged when discussing plans for the future: a) shift from hopelessness to ownership and optimism c) a call to serve community; d) planning for healthy families; and e) resilience. We discuss each theme and present supporting quotes in Table 1.

**Table 1:** Future plans.

Themes	Key words	Quotes
**Shift from hopelessness to ownership and optimism for the future:** All YPMs who described past experiences of hopelessness and thoughts of suicide now enthusiastically shared 5- and 10-year plans	“I still have a future” “I’m not alone” “I can” “hope”	*This experience has really changed much*. *Like I said*, *when I look back I’m one person who never used to think about the future*. *I would just say today—I would even tell myself that "Ah*, *why am I even going to school*? *After when someone has HIV then will just find yourself you’re dead now*.*" Now I know that someone with HIV is also able to lead a normal life as long as the person is adhering and paying attention*, *following the doctor’s instructions*, *and I also have the right to think ahead and think about my future*, *and the most important thing that can also help me is always love myself and pay more attention to myself*, *not to what people say*. *Mmm*. *Or if that negative thought would come in I would just tell that negative thought "Ah*, *move back*!*" (Female YPM)*
		*Just like I always keep on telling the participants to say "You can become somebody who you want to become in the future despite your HIV status" I also keep on pushing to say "Yes*, *I can become the person who I want to become*.*" (Female YPM)*
		*It makes me think that I can do anything*. *Because I used to ignore myself*. *Thinking*, *no*, *I couldn’t do anything*. *No*. *That’s that way and do this*. *No*. *After that training*, *after meeting others*, *and so*, *then*, *I can do everything now*. *Yes*. *(Male YPM)*
**A call to serve community:** Almost all YPMs also described new goals to serve their communities and other people living with HIV as doctors, midwives, pharmacists, and community leaders	“help” “inspire” “educate”	*My professional goals*, *to inspire*. *Yeah*. *To inspire*, *mostly*. *Because mostly*, *in the world—in this world*, *you find out that we lack people to inspire us*. *That’s why we fall into the wrong traps*. *Yeah*. *We lack people to teach us the right ways*, *people who have been through that*. *Like*, *to be a right channel of me giving my experience to someone so that it can be helpful to that person*. *…I really want to be*, *like*, *the light of the world*. *So if there is anyone who needs someone to inspire*, *I’ll be there for them*. *Yeah*. *I’ll be there for them*. *(Male YPM)*
		*It’s just removed a gift out of me… it’s more like a calling*. *(Male YPM)*
		*…being an ambassador for the adolescents*, *changing negative thinkers to positive thinking… standing in the gap for the HIV positive living*, *those who are being discriminated and stigmatized…Not just in Zambia*, *but in different countries*. *(Female YPM)*
**Planning for healthy families:** All YPMs shared how they want to get married and have healthy, HIV-negative children, a wish that many had not realized was possible until Project YES!	“negative babies” “God willing” “family”	*Back then*, *I was even afraid to have a family…*.*I remember I reached to a level of not having a child in my entire life*, *because I would just affect that child in my state*. *But now … I’ve got motivated*. *Whereby I can have a child*. *As long as I’m adhering to my medication*, *it will be fine and well*. *It will be HIV-free*. *…It feels nice—I want to be called daddy*, *a real father*. *Of which my son*, *he will not be in my state and my daughter not being in my state but be negative*. *(Male YPM)*
**Resilience:** All YPMs also expressed feelings of determination and excitement when discussing their future professional and personal plans.	“determination” “resilience” “great faith” “hardworking” “not a person who gives up easily” “nothing will stop me”	*I feel very confident*, *because I know that I can do it*. *Looking at the power–right now*, *I may not have anything to support me*, *as in*, *to stand with me*, *but I know that something will come up definitely*. *It is through my hard working*, *and being determined*. *Determination and having the resilience… If I sit down at the foot of the mountain and start crying that this mountain is just too high to go up*, *then I won’t do*, *and I won’t achieve anything*. *But if the mountain is too high*, *I’ll have to climb it*, *until [I reach] the top of its mountain*. *(Female YPM)*

### “I’m giving myself as an example”: The value of shared experience and mentoring skills to connect, build trust, and link to needed care

YPMs related to many of the challenges their clients faced, especially experiences related to stigma and ART adherence. Almost all YPMs discussed ways in which stigma can affect a person’s mental state and medication adherence, including missing doses and clinic appointments because you do not want other people to see you. A few shared strategies they discussed with clients for taking medications covertly, such as keeping the drugs in a plastic bag that doesn’t make noise like a bottle. Their own life experiences with these challenges informed how they supported with clients:

*I’m giving, like, true life examples and not like just talking maybe from a booklet or from anywhere, but I’m giving myself as an example… you’re helping someone from true life experiences, some things you’ve gone through already. (Male YPM)*

Many YPMs also saw being similar in age as an advantage when interacting with clients. As one YPM shared, being a youth allowed her clients to talk more openly about their challenges:

*They may not be comfortable sharing everything with you parents*, *yeah*, *but they’ll be so comfortable being with their fellow young people to talk the challenges*. *Even if you keep the child so nice*, *the child may have problems*, *and that child can’t share with you*, *but want to make space for these young youths to be found together and talk about what’s really affect[ing] them and their way forward*. (Female YPM)

When asked what skills a good YPM needs, all informants emphasized the importance of listening without judgment to create a foundation of trust with clients. More than any other skill, the ability to spend time and attention with each client was paramount. One YPM described what he means by “good listener”:

*A good listener is someone who should always hear our clients, not always talking, or maybe condemning or criticizing…To be a good listener is being able to acknowledge that you’re not always there to teach or maybe educate, but you’re also there to listen to others. (Male YPM)*

Several YPMs explained that paying attention to the client–“eye contact, active listening”–demonstrates to the client that what they have to share is important to you. This helped to establish an environment in which clients felt they could speak freely, ask questions, and disclose sensitive personal experiences, such as experiences of loss and grief and sexual activity.

Several YPMs explained that it took some clients more time to open up about sexual activity and did so only after deciding that the YPM was the “right person to disclose to”. One YPM shared how he used these conversations to promote condom-use and encouraged his clients to see the clinic as a resource rather than as a threat:

*The relationship which we have built between me and my clients—you see this peer mentoring really helps a lot. Yeah, because, for example, the reason why they came to me, like, maybe to ask for “Maximums”… They feel pushed down whereby they can’t get condoms. Like, maybe the person who will be giving those condoms will look down on them… I even encourage them, “No, those are trained professionals.” Yeah, “So, whenever you need them, the condoms, this and that, you can simply come at the clinic and you can get access.” Then they said, “Oh, okay. Oh, certain”–they’re receiving that–“Okay, we was afraid,” (Male YPM)*

As this quote reflects, YPMs recognized connecting AYA clients to needed services at the clinics as an important part of their role. They were keenly aware of the boundaries of their positions and understood when to link clients to HCPs for more complex or clinical challenges, such as specific questions about medications, contraceptives, or experiences of violence.

*Whenever he had the problem, [the client] could call me…[they ask] “Can they assist me or answer me this question?” And if the question is so much deep, I would say, “If you are free tomorrow, come at the facility. We’ll sit [with the] HCP together. Because not all the answers that I can give you.” (Male YPM)*

YPMs emphasized that while Project YES! is a great start, many AYA living with HIV continue to face stigma and misinformation and do not have hope for their futures. There was a shared sense among the YPMs that they had only just begun, and to stop now would mean that many would be left without needed information and support–“we have opened up their minds… we just need to continue.” As one male YPM said, “If this program could continue, I could say I could have zero viral load in every youth in Zambia.”

## Discussion

These data from Project YES! YPMs provide critical insight into the success of a youth-implemented HIV program. While the primary analysis found that Project YES! impacted internalized stigma across all participants and increased VS among AYA at the children’s hospital [24], this qualitative analysis reveals the impact that Project YES! had on the YPMs themselves and what YPMs view as the critical components of a successful peer mentoring program. This insight is all the more relevant because despite an international call for youth engagement in HIV care and prevention efforts [10,19,21,22], few models provide details on the mechanisms of how to implement a successful peer mentoring program in this population [27]. The CDC has included Project YES! in the Compendium of Evidenced Based Interventions (EBI) as a medication adherence and structural EBI.; thus, understanding the experiences of the YPMs implementing this EBI becomes critical for scale up in other contexts [25].

Project YES! YPMs identified two essential components of a peer mentoring program serving youth living with HIV. First, programs should train HIV-positive youth to be YPMs, since the shared experience of living with HIV establishes a foundation of trust between YPM and client that facilitates supportive, positive behavior change. Project YES! YPMs frequently referenced how they were able to connect with clients due to their ability to personally relate to clients’ challenges (e.g. medication adherence, stigma) and their perceived responsibility to be positive examples of what is possible with consistent, healthy practices. Programs such as the Restless Development project in Lusaka [28], Mothers2Mothers in South Africa [29], and the Zvandiri program in Zimbabwe [30] have similarly engaged peers to connect over shared experiences and health behavior role modeling. Project YES! findings reinforce the idea that peer connection may be particularly powerful among this young adult population.

Second, investment in professional training and supportive supervision impacts YPM development and their ability to support their youth clients in significant ways. Many YPMs identified the PST as the start of a period of learning and growth. The Project YES! TCBS designed interactive group activities to encourage each YPM to ask questions, challenge myths and misunderstandings, practice communication skills and boundary setting, and explore lessons through the lens of their own experiences. During interviews with several YPMs, they emphasized the importance of this kind of training and ongoing support to prepare one to share one’s own experiences for the purpose of supporting others.

In other peer support programs among youth living with HIV in SSA, such as the CATS Zvandiri Trial [30], youth implementers raised concerns about deductive disclosure (unintentional disclosure based on one’s job) as they worked in the community [31,32]. Project YES! YPMs worked in clinic settings as lay workers. As described in their interviews, YPMs valued the training they received to share their experiences as youth living with HIV to support, engage and empower their clients. In fact, the YPMs were actively involved in determining the process for disclosing their own HIV status with clients. For these reasons, the issue of deductive disclosure was not an identified limitation.

There are limitations to this analysis. First, interviews did not include questions about financial compensation which is a unique component of the Project YES! peer mentoring model. Of eight studies in a systematic review of “peer education” programs that discussed compensation, one provided no financial compensation and the other seven only provided reimbursements for time and travel [27]. Interviews with CATS from the Zvandiri trial specifically identified insufficient stipend pay as a limitation to program sustainability [31]. Project YES! YPMs pay was commensurate with that of a lay health worker, which may have contributed to the professionalism of the role and success of the model. However, these aspects were not discussed during interviews. Part of Project YES! supportive supervision involved guiding the YPMs through their first experiences negotiating contracts and receiving regular paychecks.

YPMs also did not receive the pre- and post-intervention viral load testing that youth participants in the main study received, which could have further validated their described adherence behavior changes over the course of the project. Furthermore, while our analysis did not reveal any differences in key themes by gender or age, these are factors that should be explored in larger samples as peer mentoring through Project YES! expands.

It is valuable to reflect on any potential influence an interviewer may have on the data collection process. While it is possible that because YPMs knew the interviewer ahead of time it may have influenced how they responded during the interviews, it is also possible that YPMs may have been more hesitant to discuss sensitive topics and personal experiences with someone they did not know. YPMs were assured that both positive and negative feedback was welcome and that everything they shared would help us improve the program for the next group, and YPMs did share concerns and constructive feedback with the study team throughout implementation. Additionally, we employed reflexive methods, such as discussing preliminary results with the YPMs, to limit potential bias. As this EBI is scaled up, it will be valuable to learn from YPM experiences and further examine how experiences among future YPM may change over time or differ by age and gender.

Lastly, this analysis included all eight YPMs employed with the Project YES! pilot program. While this sample size is appropriate for a homogenous sample [33] and investigators felt saturation was reached on key themes [34], we would like to see future studies explore these concepts–along with others not included in our study mentioned above–within a larger intervention.

These data have real implications for task shifting in overburdened clinics. By working with medical staff, YPMs can help address clinical human resource shortages that many low and middle income settings face [35,36]. Project YES! YPM responses reflect a keen awareness of the boundaries of their roles and a desire to improve connections between AYA clients and the clinics. This is particularly valuable considering many youth believe clinic staff do not understand what it is like to live with HIV, fear stigma, judgment, and reprimand, and therefore do not seek care [37–39]. Furthermore, employment among youth in SSA is low [40]. Within this context, YPMs have what HCPs do not–shared experience and time.

## Conclusions

Well-trained YPMs are able to communicate and support AYA clients living with HIV in unique and important ways due to their shared experiences with HIV. The Project YES! capacity-building process of becoming YPMs empowers youth to see themselves as leaders and envision healthy, productive futures. Young people living with HIV are an untapped human resource in efforts to end the HIV epidemic. Investments in this critical population have the potential to transform communities and make real impacts on the lives of people living with HIV around the world.
